# A General's Criticism

**Published:** 1919-09-20

**Authors:** 


### A General's Criticism.

A distinguished Commander of a Territorial Division used to say that his complaint of the irregular officer, whether Territorial or temporary, was that he thought he had finished his duty by the sick soldier when he had diagnosed the complaint and ordered the treatment—that he did not realise it to be his further duty to see that it was possible to give the treatment laid down, and also to make sure that it was carried out. He used further to add that the most distinguished specialist from Harley Street was of little value if he did not do these latter things. The Divisional Commander used especially to peg away at the medical officers in his field ambulance to be sure that they supervised the cooking and serving of the rations or medical comforts which had been ordered for the patients.

### Its Justification.

We believe that there is a considerable ground for this General's criticisms—and, indeed, he was seldom wrong. When a doctor has to go beyond such generalities as a "light but nourishing diet," or "keep him on farinaceous food," and to enter into details of the menu under such circumstances, he usually finds himself among difficulties. We believe that this deficiency starts when he is a student or young house surgeon. We are further of opinion that the jealousy of the nursing staff of our big hospitals is largely responsible for this. Let us consider what would be said if the house physician inquired into the way that the beef-juice was prepared, or wished to supervise the making of an egg flip by the probationer new to his ward. We fear he would either be told not to interfere with business that was not his own or else accused of making the flip an excuse for a conversation with the probationer in the serving pantry. And yet it is most important for his present patients that those things should be served properly, and for his future patients that he should be able if need be to give directions how they can be prepared to someone who has not had the advantages of a nurse's training.

### Knowledge of Food Prices.

A result is that many a man is dependent upon certain excellent but very expensive dietetic delicacies about which he knows from the advertisements and samples which have been sent to him. We know of one distinguished physician who advised a particular brand of essence of chicken at half-a-crown a tin to be spread on toast and given to the child of a young couple who were trying to keep up appearances with an assured income of £3 a week. To order Swiss milk or golden syrup at the present time for the children of some of our poor is as useless as the advice given above to the middle-class household.

